# Noise in the operating room coincides with surgical difficulty

**DOI:** 10.1093/bjsopen/zrae098

**Published:** 2024-10-16

**Authors:** Sarah Peisl, Daniel Sánchez-Taltavull, Hugo Guillen-Ramirez, Franziska Tschan, Norbert K Semmer, Martin Hübner, Nicolas Demartines, Simon G Wrann, Stefan Gutknecht, Markus Weber, Daniel Candinas, Guido Beldi, Sandra Keller

**Affiliations:** Department of Visceral Surgery and Medicine, Bern University Hospital, University of Bern, Bern, Switzerland; Department of Visceral Surgery and Medicine, Bern University Hospital, University of Bern, Bern, Switzerland; Department of Visceral Surgery and Medicine, Bern University Hospital, University of Bern, Bern, Switzerland; Institute of Work and Organisational Psychology, University of Neuchâtel, Neuchâtel, Switzerland; Department of Work and Organizational Psychology, Institute of Psychology, University of Bern, Bern, Switzerland; Department of Visceral Surgery, Lausanne University Hospital CHUV, University of Lausanne (UNIL), Lausanne, Switzerland; Department of Visceral Surgery, Lausanne University Hospital CHUV, University of Lausanne (UNIL), Lausanne, Switzerland; Department of Surgery, Triemli Hospital, Zurich, Switzerland; Department of Surgery, Triemli Hospital, Zurich, Switzerland; Department of Surgery, Triemli Hospital, Zurich, Switzerland; Department of Visceral Surgery and Medicine, Bern University Hospital, University of Bern, Bern, Switzerland; Department of Visceral Surgery and Medicine, Bern University Hospital, University of Bern, Bern, Switzerland; Department of Visceral Surgery and Medicine, Bern University Hospital, University of Bern, Bern, Switzerland

## Abstract

**Background:**

Noise in the operating room has been shown to distract the surgical team and to be associated with postoperative complications. It is, however, unclear whether complications after noisy operations are the result of objective or subjective surgical difficulty or the consequence of distraction of the operating room team by noise.

**Methods:**

Noise level measurements were prospectively performed during operations in four Swiss hospitals. Objective difficulty for each operation was calculated based on surgical magnitude as suggested by the Physiological and Operative Severity Score for the enUmeration of Mortality and Morbidity (POSSUM), duration of operation and surgical approach. Subjective difficulty and distraction were evaluated by a questionnaire filled out by the operating room team members. Complications were assessed 30 days after surgery. Using regression analyses, the relationship between objective and subjective difficulty, distraction, intraoperative noise and postoperative complications was tested.

**Results:**

Postoperative complications occurred after 121 (38%) of the 294 procedures included. Noise levels were significantly higher in operations that were objectively and subjectively more difficult (59.89 *versus* 58.35 dB(A), *P* < 0.001) and operations that resulted in postoperative complications (59.05 *versus* 58.77 dB(A), *P* = 0.004). Multivariable regression analyses revealed that subjective difficulty as reported by all members of the surgical team, but not distraction, was highly associated with noise and complications. Only objective surgical difficulty independently predicted noise and postoperative complications.

**Conclusion:**

Noise in the operating room is a surrogate of surgical difficulty and thereby predicts postoperative complications.

## Teaser

Intraoperative noise is a strong surrogate for surgical complications. The association between noise and postoperative complications is confounded by the objective surgical difficulty, which was the sole independent predictor of both intraoperative noise and postoperative complications. Thus, noise serves as an indirect marker of the challenges inherent in difficult operations, indicating that more difficult operations are usually noisier.

## Introduction

Environmental noise is considered to be a common stressor of healthcare workers^1,2^. Operating rooms are places where tasks requiring high levels of concentration are performed and effective communication is crucial. However, intraoperative noise has been shown to regularly surpass 55 dB(A) (A-weighted decibels)^3–6^, significantly exceeding the World Health Organization's (WHO) recommended limit of 35 dB(A) for operating room settings^7^. High levels of intraoperative noise have the potential to be a source of distraction for the operating room team and may interfere with surgical performance: noise in the operating room can impede effective communication among staff, potentially threatening patient safety, particularly during crucial phases of the procedures. It is also associated with increased stress and fatigue among the operating room team members^3,5,8–11^.

Besides the detrimental impact of noise on the operating room staff, there is evidence suggesting an association between intraoperative noise and postoperative complications, including but not limited to, surgical site infections (SSI)^12,13^. During operations on patients who developed SSI, intraoperative noise was significantly higher, especially during wound closure^12,13^. The overall complication rate following paediatric operations was reduced after the implementation of workplace regulations for noise reduction^14^, supporting the premise that higher intraoperative noise might significantly impact patient outcome. Although noise in the operating room is recognized as an important threat to patient safety, a recent systematic review found only four studies that empirically measured the impact of noise on patient outcome^15^. In the same systematic review, only six studies focused on the effect of noise on teams in the operating room as well as their perception of noise, indicating that more research is needed to confirm the impact of noise on patient outcomes.

Further, the mechanisms underlying the associations between noise and patient outcomes have not yet been empirically investigated. The authors of a review and meta-analysis on distractions, interruptions and disruptions in the operating room came to the conclusion that research is lacking on the mechanisms underlying the association between distracting aspects during operations and team performance and safety; these conclusions also apply for research on noise as a distractor^16^.

Research on noise in the operating room showed that higher noise levels are caused mainly by equipment and material, suggesting that operations with more material and equipment are more likely to be loud^17,18^. Operations with more material and equipment are also likely to be more complex operations, inherently associated with higher postoperative complication rates^19^. However, to the best of our knowledge, no study to date has simultaneously assessed the effect of distractions and complexity of operations on postoperative complications.

Thus, it remains to be investigated whether the occurrence of complications after operations with higher noise levels is a consequence of operating room staff distraction, that is noise being a surrogate for team distraction, or whether complications are related to specific characteristics inherent to the surgical procedure itself, that is more staff and equipment required, making noise a surrogate for surgical difficulty. The hypothesis of the study was that objective and subjective surgical difficulty, rather than team distraction by operating room noise, is an independent predictor of postoperative complications. The aim was, therefore, to test whether complications after noisy operations are the consequence of higher surgical difficulty or higher noise.

## Methods

### Study design and participants

This study was conducted within the framework of the StOP? study^20^, aimed to test the impact of the StOP?-protocol on patient outcomes. The StOP?-protocol is a short intraoperative briefing (30–90 s), performed by the surgeon leading the operation, once or several times during an operation, at the time(s) chosen by the surgeon. Its goal is to update the operating room team members about the status of the operation (St), objectives (O) for the next steps, potential problems (P) and open the floor for questions and contributions (?)^20^. In a before-after study, a 9-month baseline interval was followed by 9 months during which the StOP?-protocol was implemented; complication rates before and after the implementation of the StOP?-protocol were compared. This multicentre study was conducted in four hospitals in Switzerland between January 2015 and March 2018, and included all patients undergoing surgical procedures within the participating departments. Exclusion criteria were patient age under 18 years, presence of an SSI, operation at the same site within the preceding 30 days, outpatient procedures not requiring general anaesthesia and proctological surgery.

For a subsample of the operations included in the StOP? study, work and organizational psychologists were present to install the noise-measuring device in the operating room and to collect postoperative questionnaires on self-reported difficulty and distraction by the operating room team. Operations were selected based on the availability of the research team and the schedule of the operations. Only operations that were scheduled at least 1 day prior were included.

The study was approved by the ethical committees (leading committee: #161/ 2014). In three centres, inclusion was based on patient general consent, and in one centre the local ethical committee waived explicit patient consent and allowed inclusion of patients who did not oppose the use of their data.

### Noise measurements

A noise level measuring device VOLTCRAFT SL-451 was placed within 1.5 m of the main surgical lamp. Noise levels were registered every second in decibels dB(A). To minimize batch effect due to differences in recording technique and space, noise measurements were standardized using Z-score normalization for each hospital. Following normalization, the sound exposure level (SEL), that is total energy of a sound event normalized to a 1-s duration, was computed by converting individual noise measurements (*Li*) from dB to linear units, summing them, and then converting back to dB:


$$SEL=10\times \log_{10}\;\left(\sum \;10^{\left(\frac{Li_{z}}{10}\right)}\right)$$

In a next step, the equivalent continuous sound level (Leq), also called the time-averaged sound level, was calculated in dB. Leq is the constant sound level that, in a given time, would convey the same sound energy as the actual time-varying sound. Leq was computed by adjusting the SEL value according to the duration of the noise event in seconds (*T*):


$$Leq=SEL-10\times \log_{10}\;(T)$$

Leq was computed for the entirety of each operation. Additionally, a normalization technique for the duration of each operation was introduced to ensure a temporally comparable analysis across operations of varying durations. To achieve this, each operation's duration was divided into 100 equal parts, each representing 1% of the total operation time. Then, the Leq for each 1% segment was calculated. The selection of 1% increments is justified by the optimal balance of granularity and interpretability. While it offers detailed insight into variations in noise levels throughout operations, it avoids oversegmentation that could cloud meaningful patterns, especially in shorter procedures.

For the purposes of this article, the term ‘noise’ will be used interchangeably with ‘Leq’, signifying that whenever ‘noise’ is mentioned, it refers specifically to the calculated Leq values.

### Clinical data

Patient and surgical data were collected by trained study nurses blind to the hypotheses. Procedural characteristics were comprised of operation type, surgical approach (open or minimally invasive, converted procedures were coded as open), urgency, duration of the operation and procedure duration exceeding standard time (T-time). The T-time is the procedure-specific 75th percentile of the operation time and was taken from the National Nosocomial Infections Surveillance System surveillance report^21^. Clinical data including age, American Society of Anesthesiologists (ASA) score^22^, body mass index (BMI), sex and the occurrence of postoperative complications were gathered from patient charts. Postoperative complications were collected 30 days after surgery by trained study nurses and were classified according to the Clavien–Dindo classification^23^. The Clavien–Dindo classification is a widely used tool to objectively grade postoperative complications into five different grades from deviation from the normal postoperative course without the need for pharmacological treatment or surgical, endoscopic and radiological interventions (grade I) to death of the patient (grade V)^23^. When referring to ‘complications’ in the following work, this specifically means complications classified within the Clavien–Dindo classification ranging from grades I to V.

### Objective surgical difficulty

To evaluate the objective difficulty of the surgical procedures, a composite score that incorporated three dimensions was divised: T-time exceedance, the surgical approach and the magnitude of the procedure. The surgical magnitude was determined based on the classification suggested in the ‘Physiological and Operative Severity Score for the enUmeration of Mortality and Morbidity’ (POSSUM)^24,25^. Accordingly, the operations were classified in minor (incisional, umbilical, inguinal and femoral hernia), moderate (cholecystectomy, bariatric surgery, bowel resection, operation on peripheral vessels, thoracoscopic surgery), major (colon surgery, splenic surgery, aortic procedures, thoracotomy) and complex procedures (rectum, oesophagus, pancreatic and hepatobiliary surgery, transplantation). To combine these three categorical variables into one continuous measure, Multiple Correspondence Analysis (MCA)^26^ was employed. This method allows the analysis and visualization of relationships among multiple categorical variables in a reduced-dimensional space. Preliminary to the MCA, the relationships between surgical approach, T-time exceedance and surgical magnitude were tested using the chi^2^ test of independence. After confirming significant associations (*P* < 0.050), MCA was used to obtain a single score for objective surgical difficulty. The first dimension from the MCA, showing the strongest relationships among the variables, was used as the surgical difficulty score (*Fig. S1*). To ensure a rising score corresponded with increasing surgical difficulty, the first dimension was multiplied by −1, resulting in the final objective difficulty score. The objective difficulty score was either treated as a continuous variable or dichotomized at the mean, separating operations into higher and lower difficulty groups.

### Subjective difficulty and distraction of operating room team members

At the end of each operation, surgeons, anaesthetists, and circulating and scrub nurses responded individually to a questionnaire. Subjectively perceived difficulty of the procedure was assessed with one item: ‘how difficult was this operation for you*?*’ using a 1 to 7 scale with two opposite poles ‘easy, routine’ (1) to ‘very difficult’ (7). Feeling distracted during the operation was measured by one item: ‘during this operation, I could work in a very concentrated way’ (1) to ‘…I felt very distracted’ (7).

Depending on the analysis, the subjective difficulty and distraction were treated either as a continuous variable or dichotomized. Subjective difficulty was dichotomized according to the scale's mean into low (1–3) and high (4–7) difficulty levels. For the item on distraction, scores of 2 and above were considered to indicated distraction, as any rating above ‘no distraction’ is considered undesirable^27^.

For operations involving multiple surgeons, the mean value of the two main operating surgeons for both subjective difficulty (average measure intraclass correlation coefficient (ICC) for absolute agreement of 0.62, *P* < 0.010) and distraction (average measure ICC for absolute agreement of 0.36, *P* < 0.010) was used for analysis.

### Statistical analysis

Descriptive statistics for categorical variables were reported as numbers and proportions, and for continuous data as either median with interquartile range (i.q.r.) or mean with standard deviation (s.d.) as appropriate. Categorical data were compared with Pearson chi^2^ or Fisher's exact test. Continuous variables were compared by *t* test, Wilcoxon test or ANOVA as appropriate.

Noise was plotted over time for each percentage of the operation's relative duration and the area under the noise curve (AUC) was calculated for each procedure. Comparisons of the AUC values were conducted using the *t* test or ANOVA as appropriate.

To discern whether complications after noisy operations are attributable to either the operating room team distraction or the difficulty of the surgical procedure, a series of analyses were conducted.

First, noise AUC across dichotomized objective difficulty, subjective difficulty and distraction was compared. Then, objective difficulty, subjective difficulty and distraction as continuous variables were compared between patients with and without postoperative complications. The relationship between objective difficulty, subjective difficulty and distraction was assessed using the Pearson correlation.

To identify significant predictors of noise, a multivariable linear regression analysis adjusting for objective and subjective difficulty and distraction as continuous variables was performed.

Finally, to explore whether difficulty or distraction (as continuous variables) were related to postoperative complications, multivariable logistic regression analyses were performed, adjusting for noise, sex, age, BMI, ASA score, type of hospital and whether or not the operation took place during the intervention (StOP?-protocol) as compared with the baseline.

All analyses were performed for each profession separately (surgeons, anaesthetists, and circulating and scrub nurses). Multiple testing correction to control the false discovery rate was performed for all analyses according to the Benjamini–Hochberg method^28^. A two-sided level of significance of 0.05 was used for all analyses. All statistical analyses were performed using R (R Project for Statistical Computing, Vienna, Austria).

## Results

**Fig. 1:**
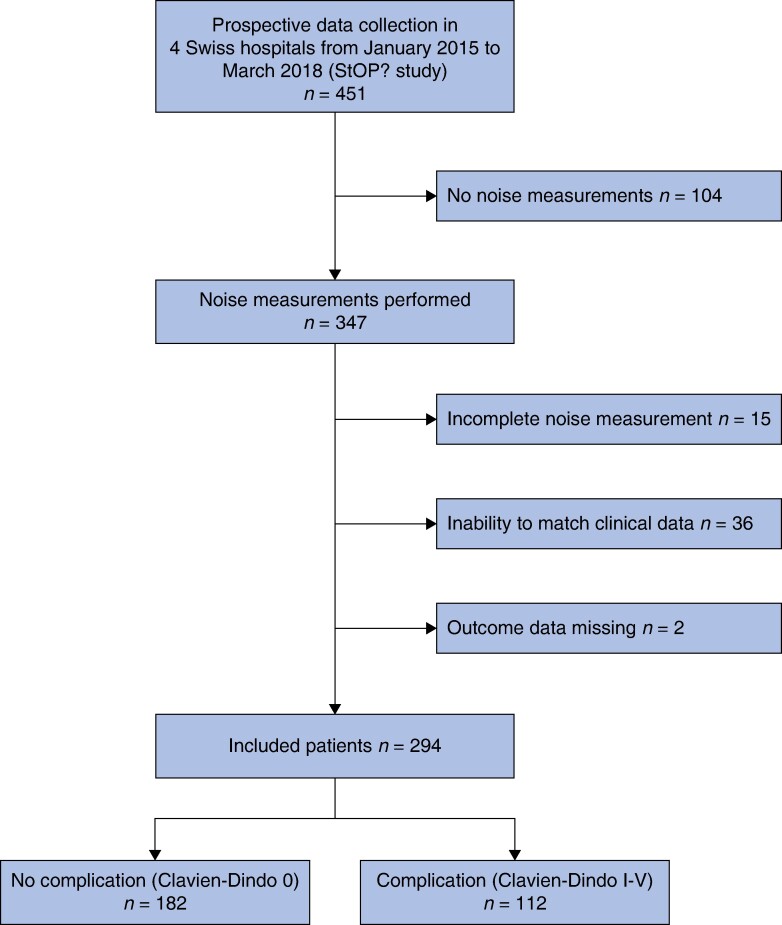
Flow chart of patient inclusion

During the StOP? study, trained psychologists were present in 451 operations. In two hospitals, a noise measurement device was installed only after a number of potentially eligible operations had already been performed, excluding 104 operations. After exclusion of incomplete noise measurements due to technical issues or human error (N = 15), inability to match clinical data and noise data (N = 36), and missing data on postoperative complications (N = 2), 294 patients were included in the analysis (*Fig. 1*). Of the operations included, 266 (90%) were abdominal surgical procedures, 12 (4%) thoracic, 9 (3%) vascular and 7 (2%) unclassified procedures (*Table 1*). A total of 167 (58%) operations were classified as major or complex; 285 (97%) were elective and 160 (54%) were open operations. Within the 30-day postoperative interval, complications occurred in 38% (N = 112) of the patients.

StOP?, status of the operation (St), objectives (O) for the next steps, potential problems (P) and open the floor for questions and contributions (?)

**Table 1:** Baseline and procedural characteristics of included patients

Variables	Overall N = 294	No complication N = 182	Complication N = 112	*P*†‡
**Sex**				0.194
Male	160 (54)	93 (51)	67 (60)	
Female	134 (46)	89 (49)	45 (40)	
Age (years), median (i.q.r)	63 (51–72)	59 (48–70)	68 (60–73)	**<0.001**
BMI (kg/m^2^), median (i.q.r)	25 (22–29)	25 (23–30)	25 (22–28)	0.399
**ASA score ≥III**	146 (50)	74 (41)	72 (64)	**<0.001**
Missing	2	2	0	
**Hospital**				**0.002**
Hospital 1	124 (42)	64 (35)	60 (54)	
Hospital 2	31 (11)	17 (9.3)	14 (12)	
Hospital 3	47 (16)	39 (21)	8 (7.1)	
Hospital 4	92 (31)	62 (34)	30 (27)	
**Main operation type**				
Hernia	41 (14)	36 (20)	5 (4.5)	
Bariatric	32 (11)	27 (15)	5 (4.5)	
Cholecystectomy	31 (11)	26 (14)	5 (4.5)	
Hepatopancreatobiliary	72 (24)	32 (18)	40 (36)	
Upper gastrointestinal	24 (8.2)	8 (4.4)	16 (14)	
Small intestinal surgery	2 (0.7)	0 (0)	2 (1.8)	
Colon	27 (9.2)	21 (12)	6 (5.4)	
Rectum	17 (5.8)	4 (2.2)	13 (12)	
Renal, adrenal	9 (3.1)	7 (3.8)	2 (1.8)	
Transplantation (kidney)	7 (2.4)	4 (2.2)	3 (2.7)	
Splenic	4 (1.4)	1 (0.5)	3 (2.7)	
Thoracic	12 (4.1)	7 (3.8)	5 (4.5)	
Vascular	9 (3.1)	3 (1.6)	6 (5.4)	
Other	7 (2.4)	6 (3.3)	1 (0.9)	
**Surgical magnitude**				**<0.001**
Minor	41 (14)	36 (20)	5 (4.5)	
Moderate	79 (28)	59 (34)	20 (18)	
Major	47 (16)	33 (19)	14 (13)	
Complex	120 (42)	48 (27)	72 (65)	
Missing	7	6	1	
Open surgical approach	160 (54)	79 (43)	81 (72)	**<0.001**
Emergency procedure	9 (3.1)	5 (2.7)	4 (3.6)	0.735
Duration of operation (hours), median (i.q.r)	2.46 (1.51–4.39)	1.95 (1.18–2.98)	4.30 (2.46–5.67)	**<0.001**
**Operation exceeded standard time (t-time)**	124 (42)	51 (28)	73 (65)	**<0.001**
Missing	1	1	0	
**Postoperative complications**				
No complication	182 (62)	182 (100)	0 (0)	
Grade I	39 (13)	0 (0)	39 (35)	
Grade II	39 (13)	0 (0)	39 (35)	
Grade IIIa&b	18 (6.1)	0 (0)	18 (16)	
Grade IVa&b	6 (2.0)	0 (0)	6 (5.4)	
Grade V	3 (1.0)	0 (0)	3 (2.7)	
Complication reported, not graded	7 (2.4)	0 (0)	7 (6.2)	

Values are *n* (%) unless otherwise indicated. †Pearson's chi-squared test; Wilcoxon rank sum test; Fisher's exact test. ‡False discovery rate correction for multiple testing. Values in bold indicate statistical significance with *P* ≤ 0.050.

**Fig. 2:**
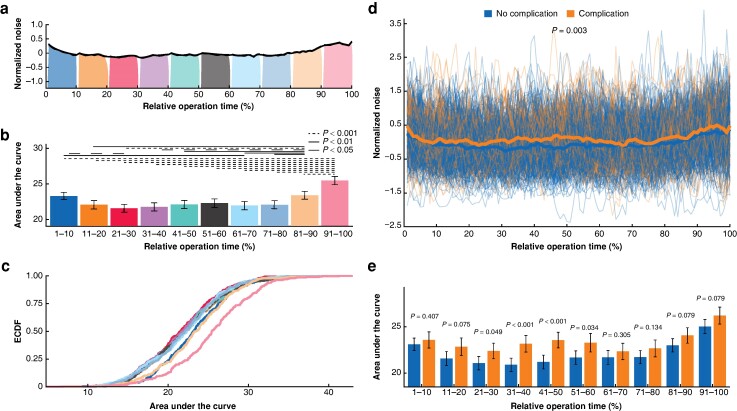
Intraoperative noise dynamics and association with complications

The averaged noise level (Leq) over the entirety of each operation ranged from 53 dB(A) to 63 dB(A), which corresponds to the noise levels of street traffic^13^. Noise levels fluctuated significantly during the operations, with the noise in the final 10% being significantly higher than in any other 10% segment (*Fig. 2a–c*).

**a** Mean of the normalized noise over time. **b** Comparison of noise AUC for every 10% of the relative duration of operation using one-way ANOVA (mean with 95% c.i.). **c** ECDF plot of noise AUC of every 10% of the relative duration of the operation. **d** Comparison of noise AUC between patients with and without complications using *t* test (individual operations plotted as thin lines, mean noise plotted as thick lines). **e** Comparison of noise AUC for every 10% of the relative operation time between patients with and without complications using *t* test. AUC, area under the curve; ECDF, empirical cumulative distribution function.

Noise levels were significantly higher during operations of patients who developed postoperative complications compared with those without complications (59.05 *versus* 58.77 dB(A), *P* = 0.004, *Fig. 2d*, *Table S1*). This effect was mostly driven by the middle part of the operation (between 21 and 60% of the relative operation time, *Fig. 2e*).

**Fig. 3:**
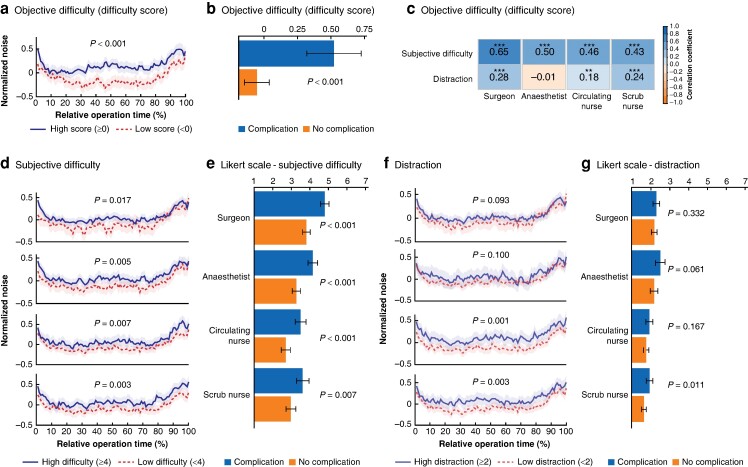
Objective and subjective difficulty association with intraoperative noise and postoperative complications

Next, whether surgical difficulty and operating room team distraction were associated with noise and complications was tested. Operations with higher objective difficulty showed significantly higher noise levels (*Fig. 3a*) and were more likely to lead to postoperative complications (*Fig. 3b*). Also, subjective surgical difficulty was highly related to objective difficulty for all team members, with correlation being strongest for surgeons, followed by anaesthetists, and circulating and scrub nurses (*Fig. 3c*). Subjective surgical difficulty was also significantly associated with noise and postoperative complications for all team members, similar to objective difficulty (*Fig. 3d–e*, *Table S1*, *Fig. S1*).

**a** Means and 95% c.i. of normalized noise over time, stratified by dichotomized difficulty score. **b** Barplot with means and 95% c.i. of objective difficulty score for patients with and without postoperative complication. **c** Pearson correlation between objective and subjective difficulty and distraction of all operating room team members. ***P value < 0.001, **P value < 0.010, *P value < 0.050. **d** Means and 95% c.i. of normalized noise over time, stratified by dichotomized objective difficulty. **e** Barplot with means and 95% c.i. of objective difficulty of the operating room members (Likert scale reaching from 1- low to 7- high) for patients with and without postoperative complication. **f** Means and 95% c.i. of normalized noise over time, stratified by dichotomized distraction. **g** Barplot with means and 95% c.i. of distraction of the operating room members (Likert scale reaching from 1- low to 7- high) for patients with and without postoperative complication.

Distraction was significantly associated with noise for both circulating and scrub nurses (*Fig. 3f*), but only scrub nurses' reported distraction showed a significant association with postoperative complications (*Fig. 3g*, *Table S1*).

The multivariable linear regression analyses revealed objective surgical difficulty as the only significant predictor of noise for all professions (*P* < 0.01, *Table 2*). Similarly, objective surgical difficulty was the sole significant predictor of postoperative complications (*P* < 0.05) (*Table 3*). The results remained consistent when substituting noise with the percentage of operation time exceeding 60 dB(A) and 70 dB(A) respectively (*Tables S1–S5*, *Fig. S2*).

**Table 2:** Multivariable linear regression model predicting normalized noise

	Surgeon N = 293			Anaesthetist N = 290			Circulating nurse N = 291			Scrub nurse N = 291		
	β*	95% c.i.	*P*‡	β*	95% c.i.	*P*‡	β*	95% c.i.	*P*‡	β*	95% c.i.	*P*‡
Intercept	0.02	−0.1,0.13	0.737	0.02	−0.10,0.13	0.793	0.00	−0.11,0.11	0.935	0.00	−0.11,0.11	0.976
StOP? performed	0.06	−0.05,0.17	0.681	0.06	−0.07,0.16	0.510	0.04	−0.07,0.16	0.531	0.07	−0.04,0.18	0.353
Objective difficulty	0.23	0.09,0.37	**0.005**	0.20	0.09,0.34	**0.003**	0.21	0.09,0.33	**0.003**	0.23	0.11,0.35	**<0.001**
Subjective difficulty	0.03	−0.11,0.17	0.737	0.09	−0.02,0.23	0.249	0.13	0.01,0.26	0.086	0.56	−0.06,0.18	0.435
Distraction	0.05	−0.07,0.169	0.681	0.06	−0.06,0.17	0.510	0.11	−0.01,0.23	0.118	0.11	0.00,0.23	0.138

^*^β, standardized β coefficient. ‡*P* value after false discovery rate correction for multiple testing. Values in bold indicate statistical significance with *P* ≤ 0.050. StOP? status of the operation (St), objectives (O) for the next steps, potential problems (P) and open the floor for questions and contributions (?).

**Table 3:** Multivariable logistic regression model predicting postoperative complications within 30 days of operation (Clavien–Dindo I–V)

	Surgeon N = 293			Anaesthetist N = 290			Circulating nurse N = 291			Scrub nurse N = 291		
	OR	95% c.i.	*P*	OR	95% c.i.	*P*†	OR	95% c.i.	*P*†	OR	95% c.i.	*P*†
Female	0.72	0.41,1.27	0.363	0.78	0.44,1.39	0.574	0.73	0.42,1.28	0.459	0.71	0.40,1.25	0.394
Age (years)	1.02	1.00,1.04	0.104	1.02	1.00,1.04	0.179	1.02	1.00,1.04	0.200	1.02	1.00,1.04	0.264
BMI (kg/m^2^)	1.00	0.95,1.05	0.977	0.99	0.94,1.04	0.656	0.99	0.95,1.04	0.752	0.99	0.95,1.04	0.849
ASA score ≥ 3	1.48	0.79,2.75	0.363	1.51	0.81,2.83	0.328	1.63	0.88,3.02	0.408	1.51	0.82,2.79	0.369
**Hospital**			0.180			0.179			0.459			0.293
Hospital 1	—	—		—	—		—	—		—	—	
Hospital 2	1.22	0.42,3.59		1.52	0.51,4.63		1.42	0.48,4.22		1.56	0.55,4.58	
Hospital 3	0.30	0.10,0.87		0.36	0.12,1.00		0.43	0.15,1.21		0.38	0.13,1.05	
Hospital 4	0.67	0.33,1.39		0.73	0.36,1.49		0.80	0.40,1.60		0.85	0.42,1.70	
StOP? performed	1.21	0.64,2.27	0.624	1.17	0.62,2.22	0.656	1.19	0.63,2.23	0.656	1.17	0.62,2.21	0.777
Normalized noise	1.00	1.00,1.01	0.516	1.00	1.00,1.01	0.656	1.00	1.00,1.01	0.656	1.00	1.00,1.01	0.742
Objective difficulty	2.28	1.37,3.86	**0.015**	2.40	1.47,4.01	**0.004**	2.54	1.61,4.09	**<0.001**	2.71	1.72,4.36	**<0.001**
Subjective difficulty	1.32	1.03,1.71	0.104	1.21	0.98,1.50	0.179	1.12	0.94,1.35	0.459	1.01	0.86,1.19	0.868
Distraction	0.78	0.57,1.06	0.239	1.22	0.99,1.51	0.179	1.09	0.81,1.47	0.656	1.27	0.94,1.72	0.293

OR, odds ratio. †*P* value after false discovery rate correction for multiple testing. Values in bold indicate statistical significance with *P* ≤ 0.050. StOP?, status of the operation (St), objectives (O) for the next steps, potential problems (P) and open the floor for questions and contributions (?).

## Discussion

This prospective, multicentric study aimed to assess how intraoperative noise is associated with the occurrence of postoperative complications, if objective and subjective surgical difficulty is taken into account. The findings show that although operations with higher mean noise have significantly more complications in the unadjusted analysis, noise does not independently predict postoperative complications in the analysis adjusted for patient- and surgery-dependent variables. Notably, objective surgical difficulty emerged as the sole independent predictor of postoperative complications. Furthermore, objective surgical difficulty was the sole independent predictor for intraoperative noise.

Sources of noise in the operating room include conversations, phone calls, monitors, alarms, technical equipment and suction^17,18^. Accordingly, higher noise levels in more objectively difficult operations might be a consequence of more operating room staff being present, case-relevant or case-irrelevant communication, more changes in staff over the course of the operation due to longer duration of the procedures, but also due to more technical equipment used (that is more instruments required and opened) and more extensive intraoperative monitoring of the patient. In addition, when background noise is higher, staff may have to communicate louder to be understood, increasing in turn the noise levels^17^. This is known as the Lombard effect and may be responsible for more complex spirals of increased noise levels in operating rooms^29^. The association between the objective level of difficulty and postoperative complications highlights inherent attributes of difficult surgical procedures, such as increased duration, invasiveness and underlying patient co-morbidities^19^. Consequently, noise during the operation may primarily reflect the challenges of difficult operations, and difficult operations inherently increase the risks of complications. The authors advocate that future work on the effects of noise in the operating room should take into account the complexity of operations, both in terms of objective difficulty and perceived difficulty by operating room staff.

While the present study identifies objective surgical difficulty as the only predictor of noise and complications, existing literature recognizes noise in the operating room as a predictor of complications^12,13^. However, it is important to note that in both cited studies, not overall noise, but higher noise at the end of the operation was related to patient complications. Similarly, a relationship between noise and team-member distraction was found for operative phases with high workload only^8^.

Although average noise may not be independently related to patient complications, noise has been found to disrupt communication and concentration^3,8^. Distractions in the operating room have negative effects on staff and procedure as well as on patient outcomes^16,30,31^. The current findings reveal a role-specific susceptibility to distraction during operations. While distraction reported by the surgeon did not show any association with noise levels or postoperative complications, distraction reported by the circulating and scrub nurses was significantly associated with noise. This higher sensitivity to noise found for operating room nurses could be related to their physical positioning in the operating room. Due to their distance to the primary surgical activity, operating room nurses may struggle more with auditory clarity, especially during noisy conditions compared with members of the surgical team working closer to the main surgeon^32^. This situation is likely exacerbated by reduced verbal communication from surgeons during intervals of high noise^8^.

The results of this study show that average noise levels during operations are most likely driven by the difficulty of the operation, and do not contribute independently to patient outcomes beyond surgical difficulty. They may, however, affect the concentration of operating room staff. In general, high noise levels increase strain. Thus, the results of this study do not contradict interventions to address noise and distractions in the operating room. Such interventions are related to reduced noise levels, reduced staff stress and also patient outcomes^14,33–35^.

This study offers valuable insights, yet acknowledging its limitations is crucial. First, the observational design limits the ability to assert causality. Furthermore, the type and source of noise, music, number of people in the operating room, movement patterns, as well as decisive or sensitive parts of the operation in which operating room staff might be more susceptible to distraction, were not recorded. In the case of music, it cannot be excluded that it may have influenced noise levels without disturbing the operating room team members, because attitudes towards music in the operating room are generally positive among staff^15^. In this complex environment, it is possible that other variables and combinations of factors, which are not accounted for in this study, positively or negatively influence operating room staff and patient outcome. Intraoperative complications, such a haemorrhage, were for example not assessed as part of this study. Additionally, impact of noise may only hold for specific types of operations. However, the sample size was too small to conduct specific analyses per operation type; future research may evaluate the impact and sources of noise during similar operations (that is using the same material and processes). Furthermore, the present analysis did not consider the varying experience levels of the operating room team members, which, alongside the objective difficulty, shape their subjective perception of difficulty. Moreover, the detailed nature of distractions and their threshold for impacting outcomes was not accounted for.

Despite these limitations, the strength of this study lies in its comprehensive approach to correlating noise levels with the objective surgical difficulty and the subjective perceptions of difficulty and distraction. The adoption of a multidisciplinary perspective identified varying levels of sensitivity to noise among different team members, offering insights that could help develop tailored strategies to mitigate the impact of noise across diverse professional groups in the operating room. Furthermore, the approach to managing the heterogeneity of the study population through normalization and the creation of a difficulty score effectively countered the variations in the data. Lastly, this study encompasses the largest cohort to date assessing the impact of noise on postoperative outcomes, thereby offering enhanced generalizability and statistical robustness.

Although the findings do not establish a direct link between distraction, noise and complications, interventions to reduce noise levels in the operating room remain relevant. It is important to acknowledge that the mere intensity of noise, measured in decibels, may not be an optimal predictor for the level of distraction. Certain sounds, regardless of their volume, may prove just as distracting due to their quality. This suggests that efforts to minimize noise should consider both the decibel level and the nature of sounds, to effectively enhance the surgical environment and team performance.

The results highlight that although noise is not a direct predictor of postoperative complications, it serves as an indirect marker of the challenges inherent in difficult operations, indicating that more difficult operations are usually noisier.

## Supplementary Material

zrae098_Supplementary_Data

## Data Availability

Research data can be accessed through the Clinical Trial Unit (CTU) of the Faculty of Medicine at Bern University and Bern University Hospital, Switzerland, via e-mail at datarequest@ctu.unibe.ch. Access to data is contingent upon meeting specific criteria determined by the institution.
