# Supplementary material for: Noise in the operating room coincides with surgical difficulty
Source: BJS Open. 2024 Oct 16;8(5):zrae098. doi: 10.1093/bjsopen/zrae098 (PMC11482277; doi:10.1093/bjsopen/zrae098)
Supplement: zrae098_Supplementary_Data [file zrae098_supplementary_data.docx]

**Noise in the operating room coincides with surgical difficulty**

Sarah Peisl^1^, Daniel Sánchez-Taltavull^1^, Hugo Guillen-Ramirez^1^, Franziska Tschan^2^, Norbert K. Semmer^3^, Martin Hübner^4^, Nicolas Demartines^4^, Simon G Wrann^5^, Stefan Gutknecht^5^, Markus Weber^5^, Daniel Candinas^1^, Guido Beldi^1*^, Sandra Keller^1^

^1^Department of Visceral Surgery and Medicine, Bern University Hospital, University of Bern, Bern, Switzerland

^2^Institute of Work and Organisational Psychology, University of Neuchâtel, Neuchâtel, Switzerland

^3^Department of Work and Organizational Psychology, Institute of Psychology, University of Bern, Bern, Switzerland

^4^Department of Visceral Surgery, Lausanne University Hospital CHUV, University of Lausanne (UNIL), Lausanne, Switzerland

^5^Department of Surgery, Triemli Hospital, Zurich, Switzerland

**Corresponding author.** Guido Beldi, MD, Department of Visceral Surgery and Medicine, Inselspital, Bern University Hospital, University of Bern, Freiburgstrasse 20, 3010 Bern, Switzerland. **ORCID ID** 0000-0002-9914-3807; **Twitter**: @BeldiGuido

**Supplementary Materials - Index**

| **Supplementary Figures and Tables** |  |
| --- | --- |
| **Table S1**: Noise and survey data, stratified by the occurrence of postoperative complications (Clavien-Dindo I-V). | *page 2* |
| **Table S2:** Multivariable linear regression model predicting percentage of operation time exceeding 60 dB(A). | *page 2* |
| **Table S3:** Multivariable linear regression model predicting percentage of operation time exceeding 70 dB(A). | *page 3* |
| **Table S4:** Multivariable logistic regression model predicting postoperative complications within 30 days of operation (Clavien-Dindo I-V), including percentage of operation time exceeding 60 dB(A). | *page 4* |
| **Table S5:** Multivariable logistic regression model predicting postoperative complications within 30 days of operation (Clavien-Dindo I-V), including percentage of operation time exceeding 70 dB(A). | *page 4* |
| **Figure S1**: Multiple Correspondence Analysis (MCA) of surgical approach, T-time and surgical magnitude. | *page 5* |
| **Figure S2:** Percentage of operation time exceeding **a**: 60 dB(A) and **b**: 70 dB(A) for patients with and without postoperative complications. **c**: Percentage of operation time exceeding 60 dB(A) and **d**: 70 dB(A) dichotomised by objective difficulty. P-value assessed with T-test. | *page 5* |

**Supplementary Figures and Tables**

**Table S1:** Noise and survey data, stratified by the occurrence of postoperative complications (Clavien-Dindo I-V).

| **Variable** | **N** | **Overall**  N = 294^a^ | **No complication**  N = 182^a^ | **Complication**  N = 112^a^ | **p-value**^b^ |
| --- | --- | --- | --- | --- | --- |
| **Surgeon - Difficulty** | 293 | 4.19 (1.44) | 3.82 (1.43) | 4.80 (1.24) | **<0.001** |
| Missing |  | 1 | 1 | 0 |  |
| **Anaesthetist - Difficulty** | 290 | 3.62 (1.51) | 3.28 (1.50) | 4.16 (1.37) | **<0.001** |
| Missing |  | 4 | 4 | 0 |  |
| **Circulating nurse - Difficulty** | 291 | 2.99 (1.67) | 2.68 (1.68) | 3.48 (1.55) | **<0.001** |
| Missing |  | 3 | 2 | 1 |  |
| **Scrub nurse - Difficulty** | 291 | 3.19 (1.83) | 2.95 (1.82) | 3.59 (1.79) | **0.007** |
| Missing |  | 3 | 3 | 0 |  |
| **Surgeon - Distraction** | 293 | 2.23 (1.00) | 2.18 (1.03) | 2.30 (0.96) | 0.332 |
| Missing |  | 1 | 1 | 0 |  |
| **Anaesthetist - Distraction** | 290 | 2.30 (1.38) | 2.18 (1.38) | 2.51 (1.36) | 0.061 |
| Missing |  | 4 | 3 | 1 |  |
| **Circulating nurse - Distraction** | 290 | 1.81 (0.98) | 1.74 (0.97) | 1.91 (0.99) | 0.167 |
| Missing |  | 4 | 3 | 1 |  |
| **Scrub nurse - Distraction** | 294 | 1.74 (0.91) | 1.63 (0.85) | 1.93 (0.96) | **0.011** |
| **Noise in dB(A)** | 294 | 59.05 (2.20) | 58.77 (2.20) | 59.52 (2.12) | **0.004** |
| **Normalised noise** | 294 | 0.05 (0.43) | 0.00 (0.43) | 0.14 (0.41) | **0.003** |
| **Percentage of operation time exceeding 60 dB(A)** | 294 | 20 (14) | 19 (14) | 22 (14) | **0.006** |
| **Percentage of operation time exceeding 70 dB(A)** | 294 | 0.50 (0.53) | 0.50 (0.53) | 0.69 (0.61) | **0.006** |
| ^a^Mean (SD) | | | | | |
| ^b^T-test, false discovery rate correction for multiple testing | | | | | |
|  | | | | | |

**Table S2:** Multivariable linear regression model predicting percentage of operation time exceeding 60 dB(A).

|  | **Surgeon**  N = 293 | | |  | **Anaesthetist**  N = 290 | | |  | **Circulating nurse**  N = 291 | | |  | **Scrub nurse**  N = 291 | | | |
| --- | --- | --- | --- | --- | --- | --- | --- | --- | --- | --- | --- | --- | --- | --- | --- | --- |
|  | **β**^a^ | **95% CI**^b^ | **p**^c^ |  | **β**^a^ | **95% CI**^b^ | **p**^c^ |  | **β**^a^ | **95% CI**^b^ | **p**^c^ |  | **β**^a^ | **95% CI**^b^ | **p**^c^ | |
| **Intercept** | 12 | 5.3, 19 | 0.003 |  | 12 | 6.3, 17 | <0.001 |  | 12 | 7.3, 16 | <0.001 |  | 15 | 10, 19 | <0.001 | |
| **StOP? performed** | 5.1 | 1.9, 8.3 | **0.003** |  | 5.0 | 1.8, 8.2 | **0.004** |  | 4.9 | 1.8, 8.1 | **0.004** |  | 5.0 | 1.8, 8.3 | **0.004** | |
| **Objective difficulty** | 4.0 | 1.5, 6.4 | **0.003** |  | 4.2 | 1.9, 6.4 | **<0.001** |  | 4.3 | 2.1, 6.4 | **<0.001** |  | 4.5 | 2.4, 6.7 | **<0.001** | |
| **Subjective difficulty** | 0.43 | -0.89, 1.8 | 0.52 |  | 1.0 | -0.10, 2.2 | 0.092 |  | 0.44 | -0.58, 1.5 | 0.40 |  | 0.22 | -0.70, 1.1 | 0.64 | |
| **Distraction** | 1.3 | -0.29, 2.9 | 0.14 |  | 0.57 | -0.54, 1.7 | 0.31 |  | 2.0 | 0.29, 3.6 | 0.027 |  | 0.77 | -1.0, 2.5 | 0.50 | |
| ^a^β = non-standardised beta coefficient  ^b^CI = Confidence Interval  ^b^p-value after false discovery rate correction for multiple testing | | | | | | | | | | | | | | | |  |

**Table S3:** Multivariable linear regression model predicting percentage of operation time exceeding 70 dB(A).

|  | **Surgeon**  N = 293 | | |  | **Anaesthetist**  N = 290 | | |  | **Circulating nurse**  N = 291 | | |  | **Scrub nurse**  N = 291 | | | |
| --- | --- | --- | --- | --- | --- | --- | --- | --- | --- | --- | --- | --- | --- | --- | --- | --- |
|  | **β**^a^ | **95% CI**^b^ | **p**^c^ |  | **β**^a^ | **95% CI**^b^ | **p**^c^ |  | **β**^a^ | **95% CI**^b^ | **p**^c^ |  | **β**^a^ | **95% CI**^b^ | **p**^c^ | |
| **Intercept** | 0.27 | -0.01, 0.54 | 0.092 |  | 0.25 | 0.04, 0.47 | 0.056 |  | 0.31 | 0.13, 0.50 | 0.002 |  | 0.42 | 0.23, 0.61 | <0.001 | |
| **StOP? performed** | 0.12 | -0.01, 0.25 | 0.092 |  | 0.11 | -0.02, 0.24 | 0.093 |  | 0.13 | -0.01, 0.26 | 0.10 |  | 0.12 | -0.02, 0.25 | 0.14 | |
| **Objective difficulty** | 0.20 | 0.09, 0.30 | **<0.001** |  | 0.21 | 0.12, 0.31 | **<0.001** |  | 0.21 | 0.12, 0.30 | **<0.001** |  | 0.22 | 0.13, 0.31 | **<0.001** | |
| **Subjective difficulty** | 0.02 | -0.03, 0.07 | 0.44 |  | 0.04 | -0.01, 0.09 | 0.093 |  | 0.02 | -0.02, 0.07 | 0.28 |  | 0.02 | -0.02, 0.06 | 0.32 | |
| **Distraction** | 0.07 | 0.00, 0.13 | 0.092 |  | 0.05 | 0.00, 0.09 | 0.084 |  | 0.06 | -0.01, 0.13 | 0.10 |  | 0.00 | -0.07, 0.08 | 0.90 | |
| ^a^β = non-standardised beta coefficient  ^b^CI = Confidence Interval  ^b^p-value after false discovery rate correction for multiple testing | | | | | | | | | | | | | | | |  |

**Table S4:** Multivariable logistic regression model predicting postoperative complications within 30 days of operation (Clavien-Dindo I-V) including the percentage of operation time exceeding 60 dB(A).

|  | **Surgeon**  N = 293 | | |  | **Anaesthetist**  N = 290 | | |  | **Circulating nurse**  N = 291 | | |  | **Scrub nurse**  N = 291 | | |
| --- | --- | --- | --- | --- | --- | --- | --- | --- | --- | --- | --- | --- | --- | --- | --- |
|  | **OR**^a^ | **95% CI**^a^ | **p**^b^ |  | **OR**^a^ | **95% CI**^a^ | **p**^b^ |  | **OR**^a^ | **95% CI**^a^ | **p**^b^ |  | **OR**^a^ | **95% CI**^a^ | **p**^b^ |
| **Female** | 0.71 | 0.40, 1.25 | 0.34 |  | 0.78 | 0.44, 1.37 | 0.55 |  | 0.73 | 0.41, 1.27 | 0.44 |  | 0.71 | 0.40, 1.24 | 0.38 |
| **Age [years]** | 1.02 | 1.00, 1.04 | 0.11 |  | 1.02 | 1.00, 1.04 | 0.20 |  | 1.02 | 1.00, 1.04 | 0.20 |  | 1.02 | 1.00, 1.04 | 0.27 |
| **BMI [kg/m^2^]** | 1.00 | 0.95, 1.05 | 0.97 |  | 0.99 | 0.94, 1.04 | 0.67 |  | 0.99 | 0.95, 1.04 | 0.76 |  | 0.99 | 0.95, 1.04 | 0.85 |
| **ASA score ≥ 3** | 1.48 | 0.80, 2.76 | 0.34 |  | 1.51 | 0.80, 2.82 | 0.33 |  | 1.62 | 0.88, 3.01 | 0.41 |  | 1.51 | 0.82, 2.78 | 0.37 |
| **Hospital** |  |  | 0.21 |  |  |  | 0.20 |  |  |  | 0.44 |  |  |  | 0.27 |
| Hospital 1 | — | — |  |  | — | — |  |  | — | — |  |  | — | — |  |
| Hospital 2 | 1.22 | 0.42, 3.62 |  |  | 1.51 | 0.50, 4.64 |  |  | 1.39 | 0.47, 4.18 |  |  | 1.55 | 0.53, 4.56 |  |
| Hospital 3 | 0.31 | 0.10, 0.88 |  |  | 0.36 | 0.12, 1.00 |  |  | 0.43 | 0.14, 1.20 |  |  | 0.38 | 0.13, 1.04 |  |
| Hospital 4 | 0.78 | 0.36, 1.68 |  |  | 0.81 | 0.38, 1.74 |  |  | 0.90 | 0.43, 1.90 |  |  | 0.95 | 0.45, 2.02 |  |
| **StOP? performed** | 1.19 | 0.63, 2.25 | 0.65 |  | 1.16 | 0.61, 2.21 | 0.67 |  | 1.18 | 0.63, 2.21 | 0.68 |  | 1.16 | 0.62, 2.19 | 0.80 |
| **Percentage of operation time exceeding 60 dB(A)** | 1.01 | 0.98, 1.04 | 0.64 |  | 1.01 | 0.98, 1.03 | 0.67 |  | 1.01 | 0.98, 1.03 | 0.68 |  | 1.01 | 0.98, 1.03 | 0.80 |
| **Objective difficulty** | 2.33 | 1.41, 3.93 | **0.009** |  | 2.45 | 1.52, 4.05 | **0.002** |  | 2.57 | 1.64, 4.10 | **<0.001** |  | 2.75 | 1.76, 4.39 | **<0.001** |
| **Subjective difficulty** | 1.32 | 1.03, 1.70 | 0.11 |  | 1.21 | 0.98, 1.50 | 0.20 |  | 1.13 | 0.94, 1.35 | 0.44 |  | 1.02 | 0.86, 1.19 | 0.85 |
| **Distraction** | 0.79 | 0.57, 1.07 | 0.26 |  | 1.22 | 0.99, 1.51 | 0.20 |  | 1.09 | 0.81, 1.47 | 0.68 |  | 1.28 | 0.95, 1.73 | 0.27 |
| ^a^OR = Odds Ratio, CI = Confidence Interval  ^b^p-value after false discovery rate correction for multiple testing | | | | | | | | | | | | | | | |

**Table S5:** Multivariable logistic regression model predicting postoperative complications within 30 days of operation (Clavien-Dindo I-V), including percentage of operation time exceeding 70 dB(A).

|  | **Surgeon**  N = 293 | | |  | **Anaesthetist**  N = 290 | | |  | **Circulating nurse**  N = 291 | | |  | **Scrub nurse**  N = 291 | | |
| --- | --- | --- | --- | --- | --- | --- | --- | --- | --- | --- | --- | --- | --- | --- | --- |
|  | **OR**^a^ | **95% CI**^a^ | **p**^b^ |  | **OR**^a^ | **95% CI**^a^ | **p**^b^ |  | **OR**^a^ | **95% CI**^a^ | **p**^b^ |  | **OR**^a^ | **95% CI**^a^ | **p**^b^ |
| **Female** | 0.70 | 0.40, 1.22 | 0.30 |  | 0.77 | 0.43, 1.36 | 0.52 |  | 0.72 | 0.41, 1.25 | 0.40 |  | 0.70 | 0.40, 1.22 | 0.35 |
| **Age [years]** | 1.02 | 1.00, 1.04 | 0.12 |  | 1.02 | 1.00, 1.04 | 0.18 |  | 1.02 | 1.00, 1.04 | 0.21 |  | 1.02 | 1.00, 1.04 | 0.28 |
| **BMI [kg/m^2^]** | 1.00 | 0.95, 1.05 | 0.98 |  | 0.99 | 0.94, 1.04 | 0.70 |  | 0.99 | 0.95, 1.04 | 0.84 |  | 0.99 | 0.95, 1.04 | 0.92 |
| **ASA score ≥ 3** | 1.49 | 0.80, 2.78 | 0.30 |  | 1.50 | 0.80, 2.80 | 0.35 |  | 1.63 | 0.88, 3.02 | 0.40 |  | 1.51 | 0.82, 2.78 | 0.35 |
| **Hospital** |  |  | 0.23 |  |  |  | 0.18 |  |  |  | 0.40 |  |  |  | 0.28 |
| Hospital 1 | — | — |  |  | — | — |  |  | — | — |  |  | — | — |  |
| Hospital 2 | 1.30 | 0.44, 3.87 |  |  | 1.52 | 0.50, 4.69 |  |  | 1.46 | 0.49, 4.40 |  |  | 1.64 | 0.56, 4.88 |  |
| Hospital 3 | 0.32 | 0.10, 0.93 |  |  | 0.35 | 0.12, 1.01 |  |  | 0.44 | 0.15, 1.24 |  |  | 0.39 | 0.13, 1.11 |  |
| Hospital 4 | 0.71 | 0.31, 1.62 |  |  | 0.70 | 0.30, 1.60 |  |  | 0.81 | 0.36, 1.82 |  |  | 0.89 | 0.39, 2.02 |  |
| **StOP? performed** | 1.21 | 0.64, 2.29 | 0.71 |  | 1.20 | 0.63, 2.32 | 0.70 |  | 1.20 | 0.64, 2.28 | 0.71 |  | 1.18 | 0.62, 2.24 | 0.88 |
| **Percentage of operation time exceeding 70 dB(A)** | 1.01 | 0.56, 1.83 | 0.98 |  | 0.90 | 0.49, 1.64 | 0.73 |  | 0.98 | 0.54, 1.76 | 0.94 |  | 1.03 | 0.57, 1.86 | 0.92 |
| **Objective difficulty** | 2.39 | 1.46, 4.01 | **0.005** |  | 2.53 | 1.58, 4.16 | **<0.001** |  | 2.64 | 1.69, 4.20 | **<0.001** |  | 2.82 | 1.81, 4.47 | <0.001 |
| **Subjective difficulty** | 1.32 | 1.03, 1.71 | 0.12 |  | 1.22 | 0.99, 1.52 | 0.18 |  | 1.13 | 0.94, 1.36 | 0.40 |  | 1.02 | 0.86, 1.20 | 0.92 |
| **Distraction** | 0.79 | 0.58, 1.08 | 0.28 |  | 1.23 | 1.00, 1.52 | 0.18 |  | 1.10 | 0.82, 1.49 | 0.71 |  | 1.29 | 0.96, 1.74 | 0.28 |
| ^a^OR = Odds Ratio, CI = Confidence Interval  ^b^p-value after false discovery rate correction for multiple testing | | | | | | | | | | | | | | | |

**Figure S1:** Results of Multiple Correspondence Analysis (MCA) of surgical approach, T-time and surgical magnitude. The scree plot shows that the first dimension accounts for the most variance among the variable categories (34.8%). The second plot illustrates the variable categories against the first and second dimension, showing that the categories distribute well along the first dimension in a manner consistent with objective surgical difficulty.


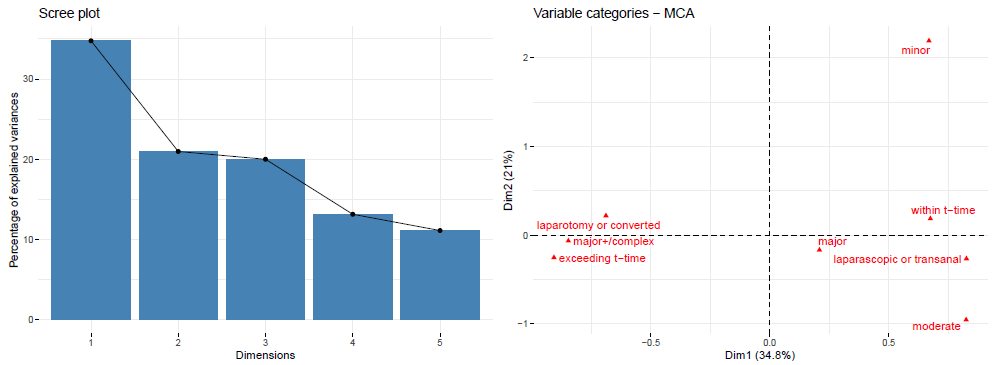


**Figure S2:** Percentage of operation time exceeding **a:** 60 dB(A) and **b:** 70 dB(A) for patients with and without postoperative complications. **c:** Percentage of operation time exceeding 60 dB(A) and **d:** 70 dB(A) dichotomised by objective difficulty score. P-value assessed using T-test.

**
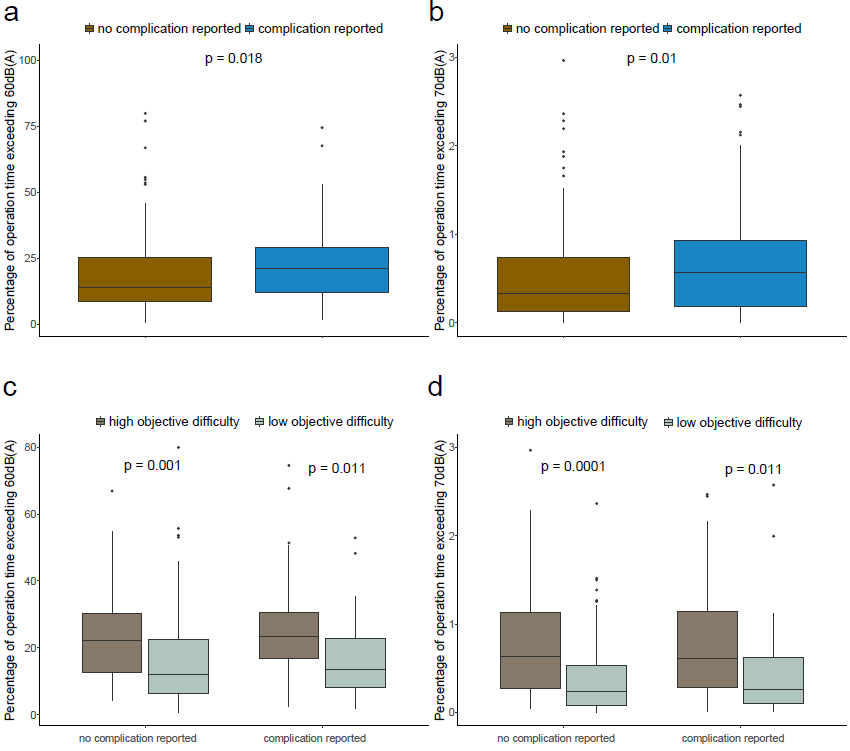
**
